# Three Polymethoxyflavones Purified from Ougan (*Citrus reticulata* Cv. *Suavissima*) Inhibited LPS-Induced NO Elevation in the Neuroglia BV-2 Cell Line via the JAK2/STAT3 Pathway

**DOI:** 10.3390/nu11040791

**Published:** 2019-04-05

**Authors:** Yue Wang, Wenjing Zang, Shiyu Ji, Jinping Cao, Chongde Sun

**Affiliations:** Laboratory of Fruit Quality Biology/The State Agriculture Ministry Laboratory of Horticultural Plant Growth, Development and Quality Improvement, Zhejiang University, Zijingang Campus, Hangzhou 310058, China; fruit@zju.edu.cn (Y.W.); zangwj@zju.edu.cn (W.Z.); jishiyu@zju.edu.cn (S.J.); caojinpingabc@126.com (J.C.)

**Keywords:** *Citrus reticulata* cv. *Suavissima*, purification, polymethoxyflavone, nobiletin, tangeretin, 5-demethylnobiletin, NO release, neuroglia, JAK2/STAT3 pathway

## Abstract

In order to establish an efficient method for separation of polymethoxyflavones (PMFs) and explore the anti-inflammatory mechanism of PMF monomers, a citrus variety rich in PMFs, Ougan (*Citrus reticulata* cv. *Suavissima*), was selected, and three monomers, including nobiletin, tangeretin, and 5-demethylnobiletin, were purified by ultrasonic-assisted extraction, solid phase extraction, and high-speed countercurrent chromatography separation. UPLC-MS was used to identify the three monomers. UPLC determined purities of 99.87% to nobiletin, 99.76% to tangeretin, and 98.75% to 5-demethylnobiletin with the standard curve method. A lipopolysaccharide (LPS)-induced NO releasing model was performed in the mouse microglia BV-2 cell line. Results illustrated that PMF monomers inhibited the NO release and the inflammation-related cytokines, including IL-1β, IL-6, and TNFα elevation. QRT-PCR revealed that PMFs alleviated LPS-induced upregulation of *iNOS*, *IL-6*, *JAK2*, *TNFα*, *IL-1β*, and *NF-κB* and LPS-induced downregulation of *IκBα*, while they did not affect *TLR1*, *TLR2*, *TLR4*, and *TLR6*. *STAT3* expression was repressed by tangeretin and 5-demethylnobiletin, but not by nobiletin. Western blot assay also showed a suppression of expression and phosphorylation of JAK2 by all three PMF monomers, while STAT3 phosphorylation was restrained by tangeretin and 5-demethylnobiletin. The mechanism was primarily verified by the JAK2 inhibitor Ruxolitinib and the STAT3 inhibitor Stattic.

## 1. Introduction

Citrus is one of the most widely distributed and consumed fruits in the world. People enjoy unique varieties of citrus because of their refreshing flavor and nutritional value. Citrus natural products, especially the flavonoids, have become a hot research topic because of their diverse biological activities [1,2,3]. Polymethoxyflavones (PMFs) are an essential type of flavonoids in citrus, characterized by more than or equal to four methoxy substitutions in the structure. Compared with other flavonoid compounds, PMFs present better bioavailability [4] and have been studied for potential anti-cancer [5], anti-diabetes [6], weight control [7], and anti-inflammation [8] activity.

Purification is one of the foundations of natural products research and the molecular sciences. Using solvent extraction, resin adsorption and separation chromatography techniques, etc., monomers can be extracted and purified from raw materials, complex mixtures, or completed compounds. Thus, their structures, physical and chemical properties, and biological activities can be determined and evaluated clearly and in detail. It is crucial to choose proper and suitable raw materials for extraction. Our previous report [9] revealed that Ougan (*Citrus reticulata* cv. *Suavissima*) is one of the citrus varieties with the highest content of PMFs. The three most abundant PMFs in Ougan are nobiletin, tangeretin, and 5-demethylnobiletin. Although the extraction and isolation of flavonoids from citrus has been studied [10], the simultaneous extraction of the three PMF monomers from Ougan fruit has not been previously reported. In this study, an efficient and manageable method was applied to separate and purify nobiletin, tangeretin, and 5-demethylnobiletin, which provided a basis for further research.

Inflammation is a stress response that occurs when tissues and cells of an organism are exposed to pathogens, harmful stimuli, or physical damage, and is closely related to the development of many diseases, such as cancer [11], atherosclerosis [12], and Alzheimer’s disease [13]. The principal pathways of inflammation include the Nuclear Factor kappa-light-chain-enhancer of activated B cells/ Inhibitor of nuclear factor kappa-B kinase (NF-κB/IκB pathway [14], the Janus kinase-2/ Signal Transducer and Activator of Transcription protein-3 (JAK2/STAT3) pathway [15], and the Toll-like receptors (TLR) pathway [16]. Ingestion of substances that inhibit inflammation in daily diets has been a research trend in the field of natural products and food research. Citrus is one of the most popular fruits in the world, and its bioactive substances have been paid close attention in anti-inflammatory research [8,17,18,19]. However, there are few reports on PMFs in the protection of microglia from inflammatory substances. Our study found that PMF monomers within a safe dose range inhibited the LPS-induced release of nitric oxide (NO) and decreased the concentrations of inflammatory factors Interleukin 1 beta (IL-1β), Interleukin 6 (IL-6), and Tumor necrosis factor alpha (TNFα), and that these effects were related to the regulation of the JAK2/STAT3 pathway. Our study offers an efficient method to purify PMFs from citrus fruit and elucidates the diverse anti-inflammatory mechanisms that natural products exert.

## 2. Materials and Methods

### 2.1. Chemicals

The chemicals used in this study are listed in Table 1. Double-distilled water (ddH_2_O) was used in all experiments. Before ultra-performance liquid chromatography (UPLC) and ultra-performance liquid chromatography-mass spectrometry (UPLC-MS) injection, the samples were filtered through a 0.22 μm membrane.

### 2.2. Materials

Ougan fruits without mechanical damage, pests, and diseases were harvested and collected in December 2016 from Lishui, Zhejiang province, China. The fruits were transported to the Fruit Science Institute of Zhejiang University in Hangzhou within 12 h. After washing, the peel of the fruit was separated and collected, then frozen in liquid nitrogen. A freeze dryer (FM 25EL-85, VirTis, Gardiner, NY, USA) was used to dehydrate the samples. Then, the samples were ground into powder and stored at −40 °C for further assays. Mouse microglia BV-2 cells were purchased from the Institute of Cells, Chinese Academy of Science (Shanghai, China).

### 2.3. UPLC and UPLC-MS Analysis of Nobiletin, Tangeretin, and 5-Demethylnobiletin

Nobiletin, tangeretin, and 5-demethylnobiletin were identified using UPLC and UPLC-MS analysis according to our previous report [9] with a modification (the detection wavelength was 330 nm). The purity of the three monomers was determined using the standard curve method.

### 2.4. Crude Extraction and Preparation of the PMF Enrichment Fraction with Solid-Phase Extraction (SPE)

Crude extraction was performed according to our previous report [20] with some modifications. 100 g of Ougan peel sample was ultrasonically extracted in 2 L of ethanol at room temperature for 30 min. The material-to-solvent ratio was 1:20 (weight/volume) and the extraction steps were repeated three times. After the mixture was centrifuged at 3900 rpm, the supernatant was collected. The ethanol was removed by evaporation under reduced pressure at 30 °C, and the precipitate was obtained for further experiments.

A Sep-pak C18 cartridge column (12 cc, 2 g sorbent, Waters Corp., Milford, MA, USA) was used for SPE for enrichment of the PMF fraction. After the samples were loaded, ddH_2_O of 50 bed volume (BV) was eluted to remove the organic acids and sugars. Then, the flavonoid components with high polarities were removed by 12 BV 35% aqueous methanol solution. Next, the PMF fraction was eluted with 100% methanol. The mixture was vacuum dried for further experiments.

### 2.5. High-speed Countercurrent Chromatography (HSCCC) Separation of Nobiletin, Tangeretin, and 5-Demethylnobiletin

HSCCC (TBE-300A, Shanghai Tauto Biotech. Co., Ltd., Shanghai, China) was used for further purification of nobiletin, tangeretin, and 5-demethylnobiletin from the PMF fraction according to our previous publication [21] with some modifications. Partition coefficients (*K* value) of the three PMF monomers in different ratios of solutions were calculated, and the *K* value was used for selecting the two-phase solvent system. Equal volumes of upper and lower phase of pre-equilibrated systems dissolved with PMF fractions were vortexed and then left to stand for 10 min for the solution to be in equilibrium. The concentrations of nobiletin, tangeretin, and 5-demethylnobiletin in the upper and lower phases were measured by UPLC and are recorded as A (*upper*) and A (*lower*), respectively. The *K* values were calculated as the ratio of A (*upper*) and A (*lower*).

Two liters of an appropriately selected solvent system were prepared and shaken well. Then, the solvent was left to stand overnight for stratification. The upper and lower phases were collected separately and ultrasonically degassed. The upper phase was used as the stationary phase, and the lower phase was used as the mobile phase. First, the stationary phase was pumped into the HSCCC separation column at a flow rate of 20 mL/min. After being filled, the countercurrent chromatograph was turned on to adjust the rotation speed to 800 rpm, and the mobile phase was pumped into the HSCCC separation column at a rate of 2 mL/min. When the mobile phase was stably discharged from the outlet, 50 mg of PMFs fraction powder dissolved in 5 mL of the lower phase solvent was injected. The effluents (1 mL/tube) were collected according to the pattern monitored by the ultraviolet (UV) chromatograph detector at 330 nm. The effluents were vacuum-dried for further detection and identification.

### 2.6. Cell Culture and NO Release Analysis

The cell culture and NO release analysis were performed according to our previous report [22]. Mouse microglia BV-2 cells were cultured at 37 °C and 5% carbon dioxide (CO_2_) in a humidified incubator with RPMI-1640 complete medium (10% fetal bovine serum). Cells in the logarithmic growth phase were seeded in 96-well plates at a density of 5 × 10^4^ cells/well and cultured for 24 h before treatment. Different concentrations of nobiletin, tangeretin, and 5-demethylnobiletin were dissolved in dimethyl sulfoxide (DMSO) and added to the fresh medium (The final volume of DMSO was 0.5% of the total medium). After 24 h of culture, 0.1 μg/mL LPS was incubated with the pretreated reagents and cells for 12 h and the NO content was determined by an NO detection kit according to the manufacturer’s instructions. DMSO was used as a solvent control.

### 2.7. Cell Viability Assay

The cell viability assay was performed according to the manufacturer’s instructions. Briefly, cells with a density of 5 × 10^4^ cells/well were plated in a 96-well plate and incubated overnight. The medium was moved, and fresh medium with the indicated reagents was added. After 72 h of incubation, the medium was dislodged, and cell counting kit-8 (CCK-8) reagent diluted with FBS-free medium was incubated with the cells for 1 h. The absorbance at 450 nm and 620 nm was detected by a microplate reader (Synergy H1, BioTek, Winooski, VT, USA). DMSO was used as a solvent control.

### 2.8. Quantitative Real-Time PCR Assay

The Quantitative Real-Time Polymerase Chain Reaction (qRT-PCR) assay was performed according to our previous report [22] with some modifications. BV-2 cells were plated onto six-well plates with a density of 5 × 10^5^ cells per well. The sequences of the primers for qRT-PCR are listed in Table 2.

### 2.9. Enzyme-Linked Immunosorbent Assay (ELISA) Assay

The ELISA assay was performed according to our previous report [22] and following the manufacturer’s instructions. BV-2 cells were plated onto 96-well plates with a density of 5 × 10^4^ cells per well. DMSO was used as the solvent control while 0.1 μg/mL LPS was used as the positive control.

### 2.10. Western Blot Assay

The western blot assay was performed according to our previous report [20]. Briefly, BV-2 cells were lysed by Nonidet P 40 (NP40) Lysis Buffer containing 1 × Halt™ Protease and a Phosphatase Inhibitor Cocktail, followed by vortex on ice using a cell crusher (JY98-IIIDN, HUXI, Shanghai, China). The supernatant was collected after centrifugation at 12,000× *g* for 10 min at 4 °C. The protein concentration was measured by an Enhanced bicinchoninic acid (BCA) Protein Assay Kit and the same amount of protein was separated on sodium dodecyl sulfate-polyacrylamide gel electrophoresis (SDS-PAGE) gels and transferred onto a polyvinylidene difluoride (PVDF) membrane (0.45 μm). The antibodies used in this study were purchased from cell signaling technology (Danvers, MA, USA). They include anti-Jak2 (dilution ratio 1:1000), anti-phospho-Jak2 (dilution ratio 1:2000), anti-Stat3 (dilution ratio 1:1000), anti-phospho-Stat3 (dilution ratio 1:1000), and anti-β-Actin (dilution ratio 1:5000). The blot complex was detected by an electrogenerated chemiluminescence (ECL) kit using the ChemiDoc™ XRS+ System (Bio-rad, Hercules, CA, USA). The amounts of protein relative to the control were quantified by Image Lab (Bio-rad, Version 3.0, Hercules, CA, USA).

### 2.11. Verification Assay Using Jak2 Inhibitor and Stat3 Inhibitor

A further verification assay was performed using the JAK2 inhibitor Ruxolitinib (Rux) and the STAT3 inhibitor Stattic. Rux (300 nM) or Stattic (10 μM) without cell toxicity (data not shown) was used to incubate BV-2 cells for 24 h before LPS treatment. After stimulation with 0.1 μg/mL LPS for 12 h, the NO release, IL-1β, IL-6, and TNFα expression, and JAK2 and STAT3 protein expression were investigated by the previously described methods.

### 2.12. Statistics

All data in this study were obtained from at least three replications. Means ± standard deviation were used for data expression. SPSS 19.0 software (IBM, Armonk, NY, USA) was employed for statistical analyses. Significant differences among different groups were analyzed using one-way ANOVA, followed by Tukey’s test at *p* < 0.05.

## 3. Results

### 3.1. Purification of Nobiletin, Tangeretin, and 5-Demethylnobiletin

#### 3.1.1. SPE

**Figure 1:**
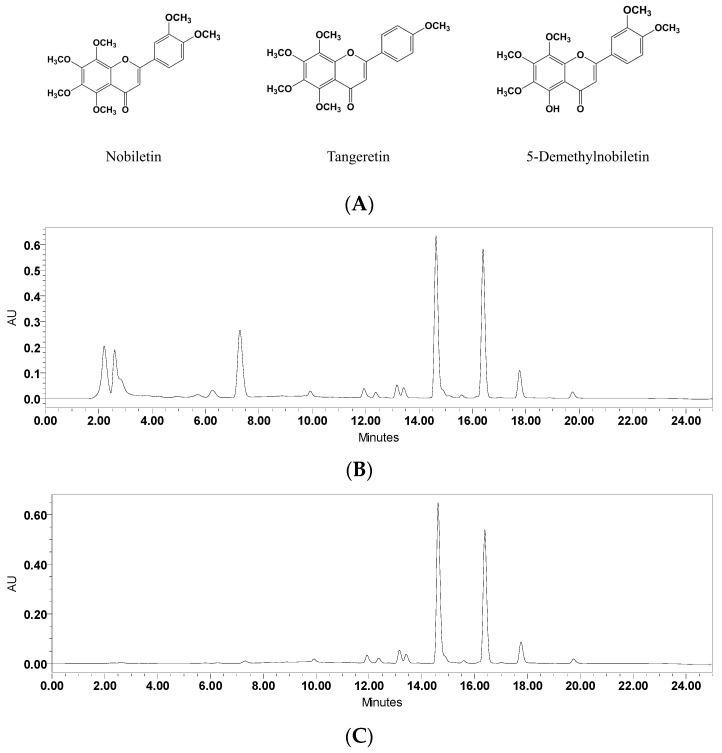
The structures and UPLC chromatograms of effects of solid-phase extraction (SPE) purification of nobiletin, tangeretin, and 5-demethylnobiletin (λ = 330 nm). (**A**) Structures of nobiletin, tangeretin, and 5-demethylnobiletin; (**B**) Crude extracts of Ougan peel; (**C**) SPE-refined polymethoxyflavone (PMF) enrichment extraction.

SPE columns were used to enrich the PMF components from crude extraction. The compounds loaded into the SPE column could be eluted sequentially according to the adsorption ability between the samples and the filler of the column. In a water–methanol mobile phase gradient elution system, compounds with greater polarity are easier to elute, while substances with a lower polarity require a higher proportion of methanol to be dissociated from the column. As shown in Figure 1, compared to the crude extraction from Ougan peel, 12 BV 35% of methanol elution was able to remove most of the components during the first 12 min.

The UPLC chromatograms proved the PMF fractions to be the flavanones in our previous report [9]. While the PMF fractions were reserved and enriched, the proportion of combined nobiletin, tangeretin, and 5-demethylnobiletin increased from 58.7% to 85.3%. The 100%-methanol-eluted PMF enrichment fraction was vacuum-dried at 30 °C while yielding a 2.01 g PMF enrichment fraction.

#### 3.1.2. High-Speed Countercurrent Chromatography (HSCCC) Purification

**Figure 2:**
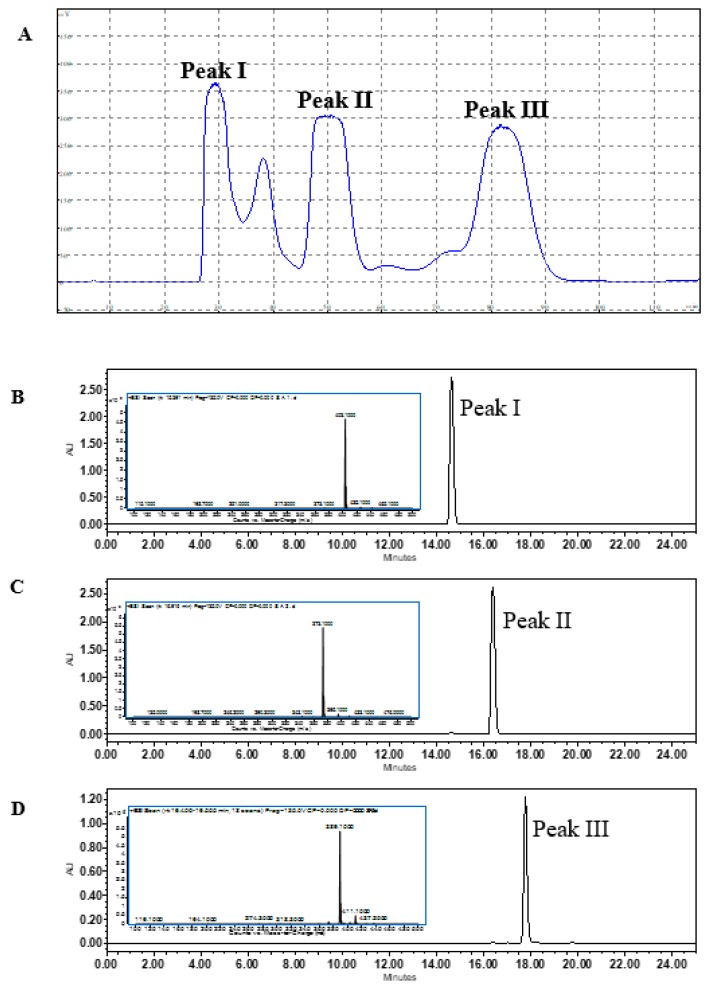
(**A**) The HSCCC separation spectrum of nobiletin, tangeretin, and 5-demetylnobiletin from the PMF fraction; (**B**) UPLC/UPLC-MS identification of purified Peak I, (**C**) Peak II, and (**D**) Peak III (λ = 330 nm).

To simultaneously separate three similar substances in the HSCCC system, it is necessary to find a suitable two-phase solvent system that produces a partition coefficient (*K* value) between 0.5 to 2 for each substance. In this study, 12 solvent systems were tested to select a proper *K* value for nobiletin, tangeretin, and 5-demethylnobiletin. As shown in Table 3, the solvent system of hexane-ethyl acetate-methanol-water (1.1:0.8:1.1:0.9) provides a *K* value for the three substances between 0.5 and 2.0 (*K_nobiletin_* = 0.55, *K_tangeretin_* = 0.78, and *K_5-demethylnobiletin_* = 1.81). The retention of the stationary phase was 57.65%. Good separation results were obtained using this solvent system (Figure 2A). Effluents of peak I, II, and III were vacuum-dried to powder at 37 °C and were weighed as W _(Peak I)_ = 16.39 mg, W _(Peak II)_ = 15.51 mg, and W _(Peak III)_ = 3.21 mg.

#### 3.1.3. UPLC-MS Analysis

UPLC-MS analysis was used for structure determination and identification of the three extracted powders. As shown in Figure 2B, the [M + H] ^+^ ion of Peak I was 403 *m*/*z*, suggesting the molecular weight number of Peak I to be 402. In the LC-MS^2^ chromatogram, fragment ions of typical products with *m*/*z* 373 [M + H − 2CH_3_] ^+^ revealed the loss of two methyl radicals, which is consistent with the previous study of nobiletin [23]. For Peak II (Figure 2C), the [M + H] ^+^ ion at *m*/*z* 373 suggests that the molecular weight number of Peak II was 372. The typical fragment ions of the LC-MS^2^ chromatogram with *m*/*z* 343 [M + H − 2CH_3_] ^+^ indicated the loss of two methyl radicals, which matches the previous report of tangeretin [24]. Figure 2D shows the [M + H] ^+^ ion at *m*/*z* 389 for Peak III, indicating that the molecular weight number was 388. The LC-MS^2^ revealed typical fragment ions of 374 [M + H − CH_3_] ^+^ and 359 [M + H − 2CH_3_] ^+^, and was consistent with the previous report of 5-demethylnobiletin [23]. After comparison with the standards, the three purified fractions were identified as nobiletin (Peak I), tangeretin (Peak II), and 5-demethylnobiletin (Peak III). The purities were determined by UPLC with the standard curve method, and the results showed that all the purities of the PMFs were above 98% (nobiletin, 99.87%; tangeretin, 99.76%; 5-demethylnobiletin, 98.75%). These powders were used for further assays.

### 3.2. Polymethoxyflavones Inhibited LPS-Induced NO Release in BV-2 Cells

**Figure 3:**
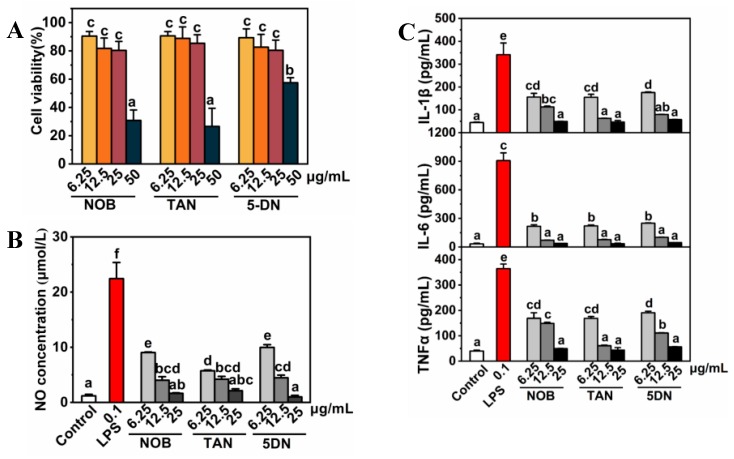
Inhibition of NO release in BV-2 cells by nobiletin, tangeretin, and 5-demethylnobiletin. (**A**) Cell viability; (**B**) NO release detection; (**C**) ELISA detection of IL-1β, IL-6, and TNFα. The lower-case letters were used to indicate the significance of numerical differences in the same graph. Columns following the same letter indicate the insignificant differences between the groups. Columns without the same letter indicate significant differences between groups (*p* < 0.05 according to Tukey’s tests). Letter ordering stated from ‘a’ and sorted by column values from small to large. 3.3. PMFs inhibit NO release by regulating the expression of JAK2 and STAT3 in BV-2 cells.

Purified PMFs monomers were used to determine the inhibition effects of NO release induced by LPS in the mouse microglia BV-2 cell line. To avoid interference caused by the cytotoxicity of PMFs, a CCK-8 assay was performed to select safe, noncytotoxic doses of the three purified monomers. The results indicated that the cellular activities under three treatments were all higher than 80% at doses between 6.25 and 25 ug/mL (Figure 3A). Therefore, the doses were used for further experiments. As shown in Figure 3B, 0.1 μg/mL LPS treatment induced the NO concentration to elevate to more than 20 times that of the control group, while all of the PMF monomers significantly prevented NO release in BV-2 cells (Figure 3B). Inflammatory factors were detected by ELISA assay, and LPS significantly elevated the cellular release of IL-1β, IL-6, and TNFα by about 300, 900, and 350 times, respectively. The PMFs treatment, within the treatment dose range, inhibited the release of inflammatory factors in a dose-dependent manner (Figure 3C).

**Figure 4:**
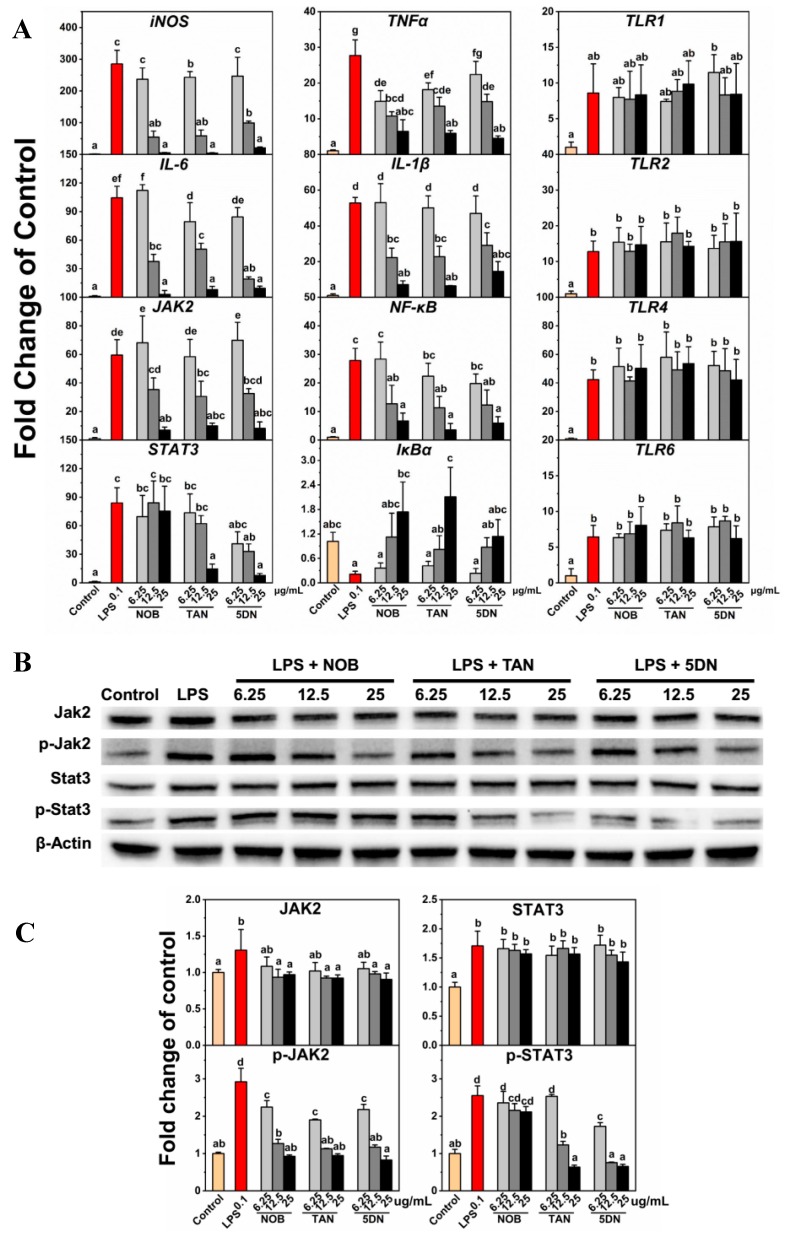
Gene and protein expression regulated by LPS and PMF monomers. (**A**) Relative gene expression detected by qRT-PCR; (**B**) Protein expression detected by western blot assay; and (**C**) Relative protein expression of Jak2, p-Jak2, Stat3, and p-Stat3. Lower-case letters were used to indicate the significance of numerical differences in the same graph. Columns following the same letter indicate the insignificant differences between the groups. Columns without the same letter indicate significant differences between groups (*p* < 0.05 according to Tukey’s tests). Letter ordering stated from ‘a’ and sorted by column values from small to large. 3.4. Preliminary verification of PMFs inhibiting the NO release mechanism.

Key gene expression for three inflammation-related pathways, including the JAK2/STAT3 pathway, the NF-κB/IκBα pathway, and the TLR pathway, was investigated using qRT-PCR. As shown in Figure 4A, PMFs restrained LPS-induced upregulation of *inducible nitric oxide synthase* (*iNOS*), *IL-6*, *JAK2*, *TNFα*, *IL-1β*, and *NF-κB* and LPS-induced downregulation of *IκBα*. For *TLR1*, *TLR2*, *TLR4*, and *TLR6*, PMFs did not affect LPS-induced gene upregulation. Previous reports have shown that tangeretin treatment can downregulate the LPS-induced gene upregulation of *iNOS*, *TNFα*, *IL-1β*, and *IL-6* [19]. Nobiletin can suppress LPS-induced NO release and was relevant in the downregulation of TNFα and NF-κB and the upregulation of IκB [17]. Tangeretin inhibited IL-1β, IL-6, and TNFα concentration in BV-2 medium and was found to be involved in NF-κB/IκBα pathway regulation [18]. Our results are consistent with previous reports and indicated that PMFs prevented the expression of the *JAK2* and *STAT3* genes. Among the three monomers, tangeretin and 5-demethylnobiletin repressed both *JAK2* and *STAT3* gene expression, while nobiletin suppressed *JAK2* gene expression but had no effect on *STAT3* expression.

The western blot assay showed that LPS induced JAK2 and STAT3 protein expression and phosphorylation, while PMFs treatment alleviated this situation. For JAK2, the protein expression was repressed by medium- (12.5 μg/mL) and high-concentration (25 μg/mL) treatments of PMF monomers and the protein phosphorylation was suppressed by all three concentrations of nobiletin, tangeretin, and 5-demethylnobiletin. For STAT3, only the three concentrations of 5-demethylnobiletin and the 12.5 and 25 μg/mL tangeretin treatments suppressed phosphorylation. Nobiletin did not affect phosphorylation, and none of the three drugs had a significant effect on the expression of STAT3 protein. The results revealed that nobiletin regulated the JAK2/STAT3 pathway by suppressing JAK2 expression and JAK2 phosphorylation, while tangeretin and 5-demethylnobileitn inhibited JAK2 expression and the phosphorylation of JAK2 and STAT3.

**Figure 5:**
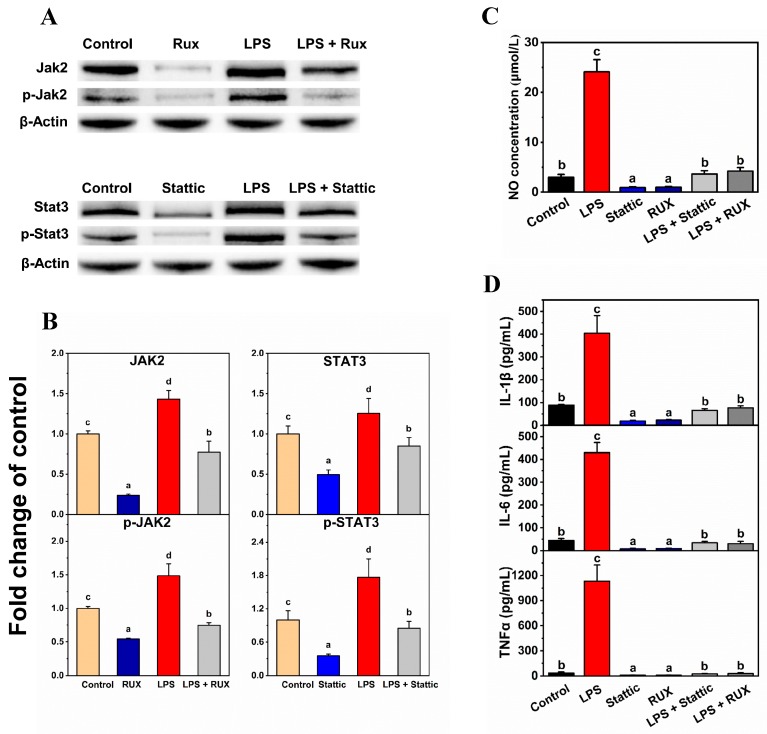
Preliminary verification by the Jak2 inhibitor and the Stat3 inhibitor. (**A**) Protein expression detected by western blot assay; (**B**) Relative protein expression of Jak2, p-Jak2, Stat3, and p-Stat3; (**C**) NO release detection; and (**D**) ELISA detection of IL-1β, IL-6, and TNFα. The concentrations of treatments were LPS (0.1 μg/mL), Rux (300 nM), and Stattic (10 μM). Lower-case letters were used to indicate the significance of numerical differences in the same graph. Columns following the same letter indicate the insignificant differences between the groups. Columns without the same letter indicate significant differences between groups (*p* < 0.05 according to Tukey’s tests). Letter ordering stated from ‘a’ and sorted by column values from small to large.

The JAK2 inhibitor Rux and the STAT3 inhibitor Stattic were used to preliminarily verify the mechanisms by which PMFs restrain NO release in BV-2 cells. As shown in Figure 5, both 300 nM of Rux and 10 μM of Stattic repressed the LPS-induced NO release and IL-1β, IL-6, and TNFα concentration in the medium, which is consistent with the results of PMFs treatments. Inhibitor treatments also respectively suppressed the JAK2 and STAT3 protein expression and the amount of phosphorylated JAK2 and STAT3. The JAK2 and STAT3 inhibitors preliminarily verified that PMFs prevented the LPS-induced release of NO in BV-2 cells by repressing the expression and phosphorylation of JAK2 and STAT3.

## 4. Discussion

Separation and purification of the monomers from the mixture make it easier to conduct in vivo and in vitro studies and to explain the mechanism of the substance, which also makes possible the determination of the primary elements in the mixture and exploration of synergy and antagonism. During the process of separating and purifying monomers, many problems will be encountered, such as a low content of substances in the raw materials, physical properties being similar between the compounds, and high costs. These problems require researchers to make efforts to choose raw materials, optimize processes, and reduce costs. Through prescreening [9], we selected a Chinese local characteristic citrus variety, Ougan, which is rich in polymethoxyflavones, as the raw material for the extraction and purification of monomers.

To facilitate the separation of PMF monomers by HSCCC, SPE columns were used for the enrichment of PMFs fractions. Flavanones were eluted and removed using a low concentration of methanol, and the PMFs were collected by elution of a high concentration of methanol. HSCCC purified nobiletin, tangeretin, and 5-demethylnobiletin with purities above 98%, and these PMFs were identified by UPLC-MS. Compared to previous studies [25,26], our purification method was able to efficiently obtain high-purity monomers with simple steps, a short separation time, and environmentally friendly operation procedures.

Lipopolysaccharides, also known as lipoglycans, are endotoxins that are used as a positive drug to induce an inflammatory reaction. It has been reported that LPS treatment could sensitize cell inflammatory pathways, activate and accumulate inflammatory molecules, promote NO production and release, and further exacerbate inflammatory responses. Previous reports have shown that citrus flavonoids, especially PMFs, could inhibit inflammation responses in various models. Citrus extractions showed the suppressing effect of NO release induced by LPS in RAW264.7 cells, and the inhibitory effect had a positive and significant correlation with the contents of nobiletin and tangeretin [27]. Nobiletin interfered with the LPS-induced production of prostaglandin E-2 and the gene expression of proinflammatory cytokines, including *IL-1α*, *IL-1β*, *TNFα*, and *IL-6* [28]. Also, nobiletin repressed the cyclooxygenase-2 (COX-2) expression and suppressed the activation of activator protein 1 (AP-1), NF-κB, and cAMP-response element binding protein (CREB) in LPS-pretreated RAW264.7 cells [29]. 5-hydroxy-3,6,7,8,3′,4′-hexamethoxyflavone prevented 12-O-tetradecanoyl phorbol-13-acetate (TPA)-induced extracellular regulated protein kinases 1/2 (ERK1/2), p38 mitogen-activated protein kinases (p38MAPK) and phosphorylated-protein kinase B (p-Akt), and iNOS activities and COX-2 expression in mouse skin, which further blocked tumor formation by reducing the tumor incidence and tumor multiplicity of papillomas [30]. The results of these studies are similar to our results. In our research, LPS-induced NO release was restrained by PMF monomers. The IL-1β, IL-6, and TNFα concentration in culture medium were suppressed by nobiletin, tangeretin, and 5-demethylnobiletin. Essential genes of three inflammatory pathways—the JAK2/STAT3 pathway, the NF-κB/IκBα pathway, and the TLR pathway—were detected. JAK2/STAT3 and NF-κB/IκBα pathway-related genes were affected by PMFs while the TLR genes did not show significant change, which revealed that the inflammatory inhibition of PMFs might not occur through the TLR pathway. Previous studies have demonstrated that, in BV-2 cells, nobiletin and tangeretin can suppress LPS-induced *iNOS*, *TNFα*, *IL-1β*, and *IL-6* gene upregulation, NF-κB protein expression, and LPS-induced downregulation of IκB [17,18,19]. However, the JAK2/STAT3 pathway has not been previously reported to be relevant to inflammatory reactions in BV-2 cells, so we further explored the mechanisms of PMFs and JAK2/STAT3 interactions. A western blot assay revealed that the three monomers had characteristics for the regulation of the JAK2/STAT3 pathway. Nobiletin repressed the expression and phosphorylation of JAK2 but did not affect STAT3. Tangeretin and 5-demethylnobiletin both prevented the expression of JAK2 and the phosphorylation of JAK2 and STAT3. Comparing the structures of the three monomers, the difference in the mechanism might be related to the number of methoxy substituents since tangeretin and 5-demethylnobiletin are pentamethoxyflavones while nobiletin is a hexamethoxyflavone (Figure 1).

Dose selection is critical in the study of the inflammatory response to a substance. A previous study reported that, among the citrus flavonoids, only nobiletin displayed a capacity of >50% to inhibit LPS-induced proinflammatory NO secretion at a concentration of 100 μM [8]. However, the cytotoxicity of nobiletin at the tested concentration was not mentioned in the experiment. In our study, we selected three concentration gradients with cell viability greater than 80%, which ensured that the experimental indicators were not caused by cytotoxicity.

Preliminary verification of the mechanisms was performed by the JAK2 inhibitor Rux and the STAT3 inhibitor Stattic. Previous reports have shown that Rux and Stattic can selectively repress the expression and phosphorylation of JAK2 [31,32,33] and STAT3 [34,35,36]. The inhibitors exhibited similar effects to PMF monomers on the restraint of LPS-induced NO release and inflammatory factor elevation in BV-2 cells. The western blot assay results also demonstrated the similar mechanism of the inhibitors and PMFs, especially the JAK2 inhibitor Rux. As for the STAT3 inhibitor Stattic, it reduced both the expression and phosphorylation of STAT3, while the PMF monomers did not affect the expression of STAT3. Since there were still differences in the patterns that affect protein changes between Stattic and PMFs, we could not conclude that the mechanism of action of the two was the same; we could only speculate about the positive correlation.

## 5. Conclusions

A distinctive citrus variety (Ougan) with a high content of PMFs was selected as the material to separate and purify three PMF monomers. PMF fractions were obtained through the ultrasonic-assisted ethanol solvent extraction and the SPE column enrichment. Three high-purity PMF monomers were obtained by further extraction of HSCCC (nobiletin, 99.87%; tangeretin, 99.76%; 5-demethylnobiletin, 98.75%). The mouse microglia BV-2 cell line was used to test the NO release induced by LPS. It was found that all three PMFs could restrain the LPS-induced NO release and reduce the expression of inflammation-related cytokines IL-1β, IL-6, and TNFα. Gene and protein expression showed that the mechanism by which PMFs suppress LPS-induced NO release might relate to the regulation of the JAK2/STAT3 pathway. The three monomers were revealed to be slightly different in their mechanisms, as nobiletin repressed JAK2 protein expression and protein phosphorylation, but did not affect the STAT3 protein. Tangeretin and 5-demethylnobiletin inhibited the expression of JAK2 and the phosphorylation of JAK2 and STAT3. The JAK2 inhibitor Rux and the STAT3 inhibitor Stattic preliminarily verified the mechanisms. Our experiments offer a preliminary basis for PMFs for further research on inflammation and neuronal cell protection and provide a simple and effective PMF purification method for in vivo and in vitro validation of other biological activities.

## Figures and Tables

**Table 1:** The chemicals used in this study.

Chemicals	Source	Address
Nobiletin standards	Sigma-Aldrich	St. Louis, MO, USA
Tangeretin standards	Sigma-Aldrich	St. Louis, MO, USA
Lipopolysaccharide	Sigma-Aldrich	St. Louis, MO, USA
methanol	Sigma-Aldrich	St. Louis, MO, USA
acetonitrile	Sigma-Aldrich	St. Louis, MO, USA
5-demethylnobiletin standards	Biobiopha Co., Ltd.	Kunming, China
Nitric oxide assay kit	Beyotime Biotechnology	Shanghai, China
RPMI 1640 medium	Gibco	Waltham, MA, USA
trypsin-EDTA	Gibco	Waltham, MA, USA
Mouse Elisa TNFα kit	R&D Systems	Carlsbad, CA, USA
Mouse Elisa IL-6 kit	R&D Systems	Carlsbad, CA, USA
Mouse Elisa IL-1β kit	R&D Systems	Carlsbad, CA, USA
Hexane	Sinopharm Chemical Reagent Co., Ltd.	Shanghai, China
ethyl acetate	Sinopharm Chemical Reagent Co., Ltd.	Shanghai, China
NP40 lysis Buffer	ThermoFisher Scientific	Waltham, MA, USA
Halt™ Protease and Phosphatase Inhibitor Cocktail	ThermoFisher Scientific	Waltham, MA, USA
Enhanced BCA Protein Assay Kit	Beyotime Biotechnology	Shanghai, China
PVDF membrane	ThermoFisher Scientific	Waltham, MA, USA
ECL kit	Service Bio	Wuhan, China
Ruxolitinib	MedChemExpress	Shanghai, China
Stattic	MedChemExpress	Shanghai, China

**Table 2:** The sequences of primers used for qRT-PCR detection.

Genes	Sequences
*GAPDH*	F: TCA ACG GCA CAG TCA AGG R: ACT CCA CGA CAT ACT CAG C
*IL-1β*	F: AGT AAG TTC CTC TCT GCA AGA GAC T R: CAC TAG GTT TGC CGA GTA GAT CTC
*IL-6*	F: GAG ACT TCC ATC CAG TTG CCT R: CAG GTC TGT TGG GAG TGG TA
*TNFα*	F: CGG GCA GGT CTA CTT TGG AG R: ACC CTG AGC CAT AAT CCC CT
*iNOS*	F: CGG CAA ACA TGA CTT CAG GC R: GCA CAT CAA AGC GGC CAT AG
*TLR1*	F: TCT CTG AAG GCT TTG TCG ATA CA R: GAC AGA GCC TGT AAG CAT ATT CG
*TLR2*	F: TCT AAA GTC GAT CCG CGA CAT R: TAC CCA GCT CGC TCA CTA CGT
*TLR4*	F: CAA GAA CAT AGA TCT GAG CTT CAA CCC R: GCT GTC CAA TAG GGA AGC TTT CTA GAG
*TLR6*	F: AAC AGG ATA CGG AGC CTT GA R: CCA GGA AAG TCA GCT TCG TC
*JAK2*	F: AAG ATG CTT TCT GGG TTG G R: ACA TTG TCT AAG AGG GAG CAG
*STAT3*	F: ACC TCC AGG ACG ACT TTG AT R: TGT CTT CTG CAC GTA CTC CA
*IκBα*	F: TAC CCC TCT ACA TCT TGC CTG T R: GTG TCA TAG CTC TCC TCA TCC TC

**Table 3:** The partition coefficient (*K* value) of nobiletin, tangeretin, and 5-demethylnobiletin in different solvent systems.

Solvent System (v/v/v/v)	Ratio	*K_nobiletin_*	*K_tangeretin_*	*K_5-demethylnobiletin_*
Hexane-ethyl acetate-methanol-water	1:0.8:1.1:1.1	0.76	1.32	2.59
Hexane-ethyl acetate-methanol-water	1:0.8:1.1:1	0.69	1.09	2.42
Hexane-ethyl acetate-methanol-water	1:0.8:1.1:0.9	0.57	0.88	2.21
Hexane-ethyl acetate-methanol-water	1:0.8:1.2:1.1	0.63	1.39	2.63
Hexane-ethyl acetate-methanol-water	1:0.8:1.2:1	0.58	1.15	2.56
Hexane-ethyl acetate-methanol-water	1:0.8:1.2:0.9	0.43	0.98	2.31
Hexane-ethyl acetate-methanol-water	1.1:0.8:1.1:1.1	0.67	1.21	2.36
Hexane-ethyl acetate-methanol-water	1.1:0.8:1.1:1	0.61	1.13	2.10
Hexane-ethyl acetate-methanol-water	1.1:0.8:1.1:0.9	0.55	0.78	1.81
Hexane-ethyl acetate-methanol-water	1.1:0.8:1.2:1.1	0.47	1.67	2.34
Hexane-ethyl acetate-methanol-water	1.1:0.8:1.2:1	0.32	1.49	1.97
Hexane-ethyl acetate-methanol-water	1.1:0.8:1.2:0.9	0.26	0.90	1.77
Hexane-ethyl acetate-methanol-water	1:0.8:1.1:1.1	0.76	1.32	2.59

## Abbreviations

AP-1	Activator protein 1
BV	Bed volume
CREB	cAMP-response element binding protein
DMSO	Dimethyl sulfoxide
ECL	Electrogenerated chemiluminescence
ERK1/2	Extracellular regulated protein kinases 1/2
HSCCC	High-speed Countercurrent Chromatography
IL-1β	Interleukin 1 beta
IL-6	Interleukin 6
iNOS	Inducible nitric oxide synthase
JAK2	Janus kinase 2
LPS	Lipopolysaccharides
NF-kappa B	Nuclear factor-kappa B
NO	Nitric oxide
MAPK	Mitogen-activated protein kinase
p-Akt	phosphorylated AKT serine/threonine kinase 1
PCR	Polymerase chain reaction
PMFs	Polymethoxyflavones
PVDF	Polyvinylidene fluoride
qRT-PCR	Quantitative Real-Time Polymerase Chain Reaction
Rux	Ruxolitinib
STAT3	Signal transducer and activator of transcription 3
TLR1	Toll-like receptor 1
TLR2	Toll-like receptor 2
TLR4	Toll-like receptor 4
TLR6	Toll-like receptor 6
TNF	Tumor Necrosis Factor α
UPLC	Ultra Performance Liquid Chromatography
UPLC-MS	Ultra Performance Liquid Chromatography-Mass Spectrometer
